# Effect of strengths-based care: community led support

**DOI:** 10.1007/s10433-025-00884-8

**Published:** 2025-10-21

**Authors:** Jonathan E. Prunty, Jinbao Zhang, Madalina Toma, Robin Miller, Julien Forder

**Affiliations:** 1 PSSRU, University of Kent, Canterbury, UK; 2 School of Social Policy, University of Birmingham, Birmingham, UK; 3 Present Address: Leverhulme Centre for the Future of Intelligence, University of Cambridge, Cambridge, UK

**Keywords:** Adult social care, Strengths-based, Asset-based, Social prescribing, Community led support

## Abstract

Strengths-based models of care are increasingly popular with policymakers, but evidence of their effectiveness is currently limited. This study examines the impact that a strengths-based care programme—community led support (CLS)—has had on new and existing clients in England. Specifically, we used a difference-in-difference approach to estimate the treatment effect of CLS on care provision, reviews, and expenditure, using the Short and Long Term (SALT) dataset published by NHS Digital (2016 to 2021). Within local authorities that implemented CLS, we found evidence of changing care pathways for new clients, including a ten-percentage-point reduction in funded care provision—though evidence for increased signposting to alternative services in this dataset was mixed. For existing clients, we found evidence of general improvements in the quality of practice, as indicated by higher ratios of planned to unplanned care reviews. These improvements were also realised without concomitant increases in expenditure rates. We believe these results can contribute towards an evidence base for CLS and for strengths-based practice more generally.

## Introduction

Strengths-based (or ‘asset-based’) models of care have received substantial policy support in the UK in recent years (Department of Health and Social Care 2018, 2019), leading to implementation in adult social care (ASC), primary care, and public health contexts (Drinkwater et al. 2019; Foot & Hopkins 2010). They are based on the critique of health and care services being deficit orientated (i.e., what an individual cannot do and the assessed risks), focused on professional opinion not on what matters to the person, and prioritising professional and service-orientated solutions over people’s own resources (e.g., their personal skills and resilience, their networks of family and friends, and supports within their local communities). This results in services potentially reducing people’s autonomy and independence alongside committing formal resources which may not be required. Given their potential to enhance care outcomes and limit healthcare expenditure, strengths-based models of care have been adopted in various countries outside the UK (Gottlieb & Gottlieb 2017; Rapp et al. 2006).

Community led support (CLS) is a strengths-based initiative led by the National Development Team for Inclusion (NDTI; ndti.org.uk; see Brown et al. 2020; Richardson & Pitts 2023). CLS and strengths-based approaches more generally provide core values and intervention strategies as a starting point with acceptance that the operationalisation of these values will differ in each specific implementation setting (Brown et al. 2020; Caiels et al. 2021; Department of Health and Social Care 2019; Miller & Whitehead 2015; Richardson & Pitts 2023). CLS is funded through individual local authorities signing up to participate and paying an annual programme fee. They receive facilitation support from the NDTI team to help them review their current processes and identify opportunities to improve. They also have access to a range of learning opportunities related to change management, impact measurement and strengths-based care models and can engage with peer support and learning through online forums and in-person networking days. In CLS, core intervention strategies include creating community access hubs in which members of the public can approach health and care professionals, reducing bureaucracy through streamlining assessment and care planning processes, and enabling greater flexibility in how social workers use budgets associated with people’s care. The blend and sequencing of these interventions will be adapted or augmented according to the needs of the local community (Miller et al. 2024). This flexibility is embraced as a core tenet of CLS, which calls itself a ‘place-based approach’ (Brown et al. 2020), but lack of consistency regarding implementation creates challenges for evaluating a framework’s effectiveness (Daly & Westwood 2018; Staudt et al. 2001).

Consequently, evidence to date of CLS effectiveness has predominantly relied on personal testimonies and smaller single-site case studies, with few attempts to evaluate the programme at a national level (Brown et al. 2017, 2020; Richardson & Pitts 2023). This pattern is not specific to CLS, but extends to strengths-based practice in general. In a recent scoping review, Caiels and colleagues (2021) concluded that despite popularity and significant investment, the evidence supporting the effectiveness of strengths-based practice was overall limited. Without robust evaluation, the ability of local areas to make evidence-based decisions regarding strengths-based interventions will remain limited. In the current analysis, we will attempt to quantify the impact that the CLS programme has had across England. Although our focus here is on CLS specifically, we believe that our results could also contribute towards an evidence base for strengths-based practice more generally.

A theory of change was developed for CLS through reviewing programme documents and interviewing members of the NDTI team and the implementation leads within local sites (Breuer et al. 2015; Miller et al. 2024). This set out the underpinning assumptions behind the implementation of CLS within local areas and the causal chains which linked activities with outcomes. Despite some variation in local contexts, there was much commonality in how the programme would adapt organisational processes and practitioner behaviours to reflect strengths-based principles—for example, changing the focus of core assessment documentation from checklists which identify areas of concern to a more conversational approach which starts from people’s aspirations and factors which enable them to have a good quality of life. Building on this theory of change, and previous reports by NDTI (Brown et al. 2017, 2020), there are several expected CLS outcomes against which its effectiveness can be gauged (see Appendix Table 6). Although some variation in CLS implementation between sites is to be expected, the outcomes listed in Table 6 should still emerge if the core values of CLS are adhered to (Brown et al. 2017, 2020). From these expected outcomes, we can generate several testable predictions about what changes should be visible and increasing once the CLS approach has been established within a region.

Firstly, CLS should affect how people access care services, and where they end up. The approach places value on high-quality, early conversations to assess a potential client’s strengths and assets, and to connect them to relevant support options based in the local community. This should result in a reduced number of people receiving funded care, alongside a concomitant increase in the number of people directed to alternative sources of support. Secondly, we hypothesise that care pathways become more efficiently organised, increasing the quality of care. For example, care that is better tailored to the needs of individuals is likely to be more effective in achieving people’s preferred outcomes. One indicator of improved quality would be a reduction in unplanned reviews of a person’s care package—or equivalently, an increase in the ratio of planned reviews (which are pre-determined and good practice) to unplanned reviews, reflecting a more proactive, rather than reactive, style of care. We should also expect increases in care quality not to come at increased costs, as they are instead driven by better use of existing resources.

## Methods

### Sample

The local authorities in England who have participated in the CLS programme at the time of the study were identified by NDTI (Appendix Table 7). These vary considerably in relation to their physical geography, population size, socio-economic status, and cultural diversity.

### Datasets

We tested the above hypotheses using administrative data provided by local authorities across England. Specifically, we used the Adult Social Care Activity and Finance Report (ASC-FR; NHS Digital 2023) and Short- and Long-Term Services (SALT; NHS Digital 2023) datasets, which include publicly available aggregated data on care access, provision, reviews and expenditure. We supplemented these datasets with local-authority level covariates published by the Office for National Statistics (ONS; ons.gov.uk), the Care Quality Commission (CQC; cqc.org.uk), and the Department for Work and Pensions (DWP; stat-xplore.dwp.gov.uk). Data extracted from these sources include population statistics, care bed availability, as well as claimant numbers for Pension Credit, Attendance Allowance, and Carer’s Allowance.

Our analysis was restricted to the six-year time window that the ASC-FR has been published at the time of writing (financial years 2016–2017 to 2021–2022). As local governments in England were restructured during this time period, we created a standardised dataset of 149 upper-tier local authorities by combining data from regions that have either merged or divided (Northamptonshire; Bournemouth, Christchurch and Poole) and by excluding two sparsely populated regions (Isles of Scilly and the City of London). Only data from older adults (aged 65 or more) were included (see Table 1).

**Table 1:** Data summary

	*Mean*	*SD*	*Min*	*Max*	*N*	*Source*
Care requests^1^	15.83	19.49	0.03	106.52	6966 (2.60)	SALT
Proportion with funded care	56.58	20.09	6.10	99.64	1769 (1.06)	SALT
Proportion of non-funded signposted	39.26	30.11	0.30	99.84	1563 (12.58)	SALT
Care reviews^2^	0.46	0.28	0.003	1.25	1783 (0.28)	SALT
Proportion planned	68.71	16.23	2.99	98.93	891 (0.34)	SALT
Care expenditure (£1000s)^1^	414.29	177.89	22.75	965.90	1780 (0.45)	ASC-FR
Care expenditure (£1000s)^2^	15.50	6.62	0.89	36.77	1780 (0.45)	ASC-FR
Attendance Allowance^1^	131.90	20.76	81.50	200.63	894 (0)	DWP
Care bed availability^1^	43.33	10.44	11.34	82.58	894 (0)	CQC
Carer’s Allowance^1^	28.48	10.98	9.48	75.91	894 (0)	DWP
Pension Credit^1^	156.22	65.31	46.17	442.82	894 (0)	DWP
Population size (1000 s)	68.75	60.93	9.41	322.78	894 (0)	ONS
Proportion over 80	26.98	1.72	21.95	31.32	894 (0)	ONS

1. per 1000 residents over 65. 2. per existing clients. Data includes older adults only (over 65s). All proportions are displayed as percentages. *N,* number of independent data points across 149 regions and 6 years, with % missingness in brackets. *N* includes multiple observations per year for: Care requests (Access Route: community and hospital, Care Destination: long-term, short-term, low-level, or non-funded), Care reviews (Review Type: planned and unplanned), and Care expenditure (Care Type: community and residential). SALT, Short and Long Term; ASC-FR, Adult Social Care Financial Return; DWP, Department of Work and Pensions; CQC, Care Quality Commission; ONS, Office for National Statistics

### Variables

*Provision.* To assess the impact of CLS on care provision, we created two proportional dependent variables. First, we calculated the proportion of total requests from new clients that resulted in local-authority funded care, whether it be long-term (9.94%), short-term (21.10%) or low-level care (17.25%). Second, of those requests not resulting in funded care (51.72%), we calculated the proportion that were signposted to other services (e.g., voluntary sector services; 50.67%). To capture differences in the *source* of care requests, the majority of which originated from either the community (75.57%) or hospital discharge services (24.43%), we also included an Access Route factor (community or hospital) within our models.

*Reviews.* The SALT dataset distinguishes between routine planned reviews (67.77%) and unplanned reviews that resulted from a sudden change in the client’s circumstances (e.g., an acute health event; 32.23%). Of the total number of reviews for existing long-term clients (i.e. those receiving care for 12 months or more at year end), we calculated the proportion that were designated as planned.

*Expenditure.* For our cost-of-care analysis, we focused on expenses related to long-term care and used gross current expenditure (GCE) as our dependent variable. GCE is the standard fiscal metric used by NHS Digital to cover local authority spending that is not offset by client income (NHS Digital 2022). All expenditure values were converted to rates of £1000 s per 1000 residents over 65. Expenditure rate was log-transformed (natural log) prior to model-fitting to reduce skewness. A Care Type factor (community or residential) was also included to differentiate expenditure for clients based in their own homes (including home care, direct payments and supported living) from expenditure for clients based permanently in a residential or nursing home (henceforth referred to as ‘residential’). Overall, a larger proportion of long-term care costs were spent on residential care (64.62%) compared to community care (35.38%).

Descriptive statistics for each of the dependent variables used in our analyses are provided in Table 1. For continuous variables, outliers (> 99%) were replaced with the threshold values, and zero values were removed. Once proportional dependent variables were computed, ones (i.e. 100%) were also removed (see Table 1 for the percentage of missing data). Additionally, Table 1 also includes summaries of covariates included in all models to capture relevant systematic variation across regions. Of these covariates, Pension Credit, Attendance Allowance, and Carer’s Allowance are all forms of financial aid available to older people, particularly those who require a carer (Carer’s Allowance) or have disabilities (Attendance Allowance). Claimant numbers for these benefits were included as indicators of physical and financial need. The total number of care beds within a region (care bed availability) was also included as an indicator of care capacity, alongside relevant population characteristics, such as over 65 population size and the percentage of the population over 80 years of age. Aside from population characteristics, all covariates were converted to rates per 1000 residents over 65. Zero values were also removed from all covariates, and population size, Pension Credit and Carer’s Allowance were log-transformed to reduce skew.

To reduce multicollinearity between covariates, Attendance Allowance was subsequently removed from our analysis as it was highly correlated with Carer’s Allowance (*r* = 0.82) and Pension Credit (*r* = 0.70), possessing a variance inflation factor (VIF) of 5.87 (Thompson et al. 2017). After the removal of Attendance Allowance, all VIFs for covariates were less than three (*M* = 1.71).

### Analysis strategy

To assess the impact that CLS implementation had on care provision, reviews, and expenditure, we used a difference-in-difference (DiD) fixed effects modelling approach (Wing et al. 2018). This approach enables us to estimate the treatment effect of CLS, while controlling for general differences across region and time and thus any underlying variables that are not explicitly measured. Formally, we estimated the equation:

$$Y_{it} = \alpha_{i} + \delta_{t} + \beta_{k} \left( {CLS_{i} \times T_{it}^{k} } \right) + \mu^{j} X_{it}^{j} + \varepsilon_{it}$$

where ${\alpha }_{i}$ is the time-invariant fixed effect for each local authority $i$ and ${\delta }_{t}$ is the time dummy for year $t$. The coefficients, ${\beta }_{k}$, are the parameters indicating the effect of CLS in the years before and after CLS implementation. Time periods, *k*, included two pre-implementation (years − 5 to − 4 and − 3 to − 2) and three post-implementation periods (years 0 to 1, 2 to 3, and 4 +), with the year prior to implementation (− 1) used as the reference year (henceforth the five non-reference time periods will be referred to as years − 4, − 2, 0, 2, and 4). The CLS treatment effect is determined by the interaction between a CLS treatment dummy, ${CLS}_{i}$, and the dummies representing pre- and post-implementation time periods, ${T}_{it}^{k}$. We then calculated the average treatment effect on the treated (ATT) by averaging across post-implementation coefficients (Sun & Abraham 2021). The parameters, ${\mu }^{j}$, estimate effects for a vector of *j* control variables, ${X}_{it}^{j}$, included within the model. All models included the covariates listed in Table 1, but provision and expenditure models also included an additional grouping factor to account for the effect of Access Route (provision) and Care Type (expenditure) on ${Y}_{it}$. Finally, ${\epsilon }_{it}$ represents idiosyncratic error values within the model. In theory, we expect the $\beta$ coefficients for time periods prior to CLS implementation (${\beta }_{k=-2}$ and ${\beta }_{k=-4}$), to be zero, after accounting for the fixed effect ${\alpha }_{i}$ and the covariates in ${X}_{it}^{j}$—that is, being a CLS site before implementation should not be different from a non-CLS site at that time, allowing for some contamination from treatment effects at other time periods (see Sun & Abraham 2021).

Recent advances in econometric theory have suggested that standard two-way fixed effect (TWFE) regression can produce biased treatment estimates when treatment times are staggered (see Baker et al. 2022) and are not robust to the inclusion of covariates (Caetano & Callaway 2024). Considering this, alongside conventional TWFE models, we also estimated DiD models using the Sun and Abraham (2021) method (S&A), which can adequately deal with dynamic treatment effects and small sample sizes, accounting for contamination of the overall treatment effect by treatment effects from other periods (using weights derived from an auxiliary regression). For both TWFE and S&A methods, we used cluster-robust standard errors to account for correlations within regions. Standard errors were adjusted post hoc by clustering at the local-authority level (Abadie et al. 2022; Cameron & Miller 2015; MacKinnon et al. 2023). All models were estimated using the ‘fixest’ package in *R* (Berge et al. 2023).

## Results

### Selection bias

Local authorities with higher care needs may be more likely to implement CLS. To explore potential selection bias, we followed Stokes and colleagues’ (2019) approach, using logistic regression analysis to examine factors associated with CLS adoption. The analysis showed that none of the covariates were statistically significant predictors of CLS implementation at the 5% level. Additionally, t-tests assessing balance in covariates before CLS implementation (Appendix Table 9) indicated no significant differences between CLS and non-CLS sites. Taken together, these results suggest observed characteristics were balanced and there was no evidence of selection bias.

### Care provision

**Fig. 1:**
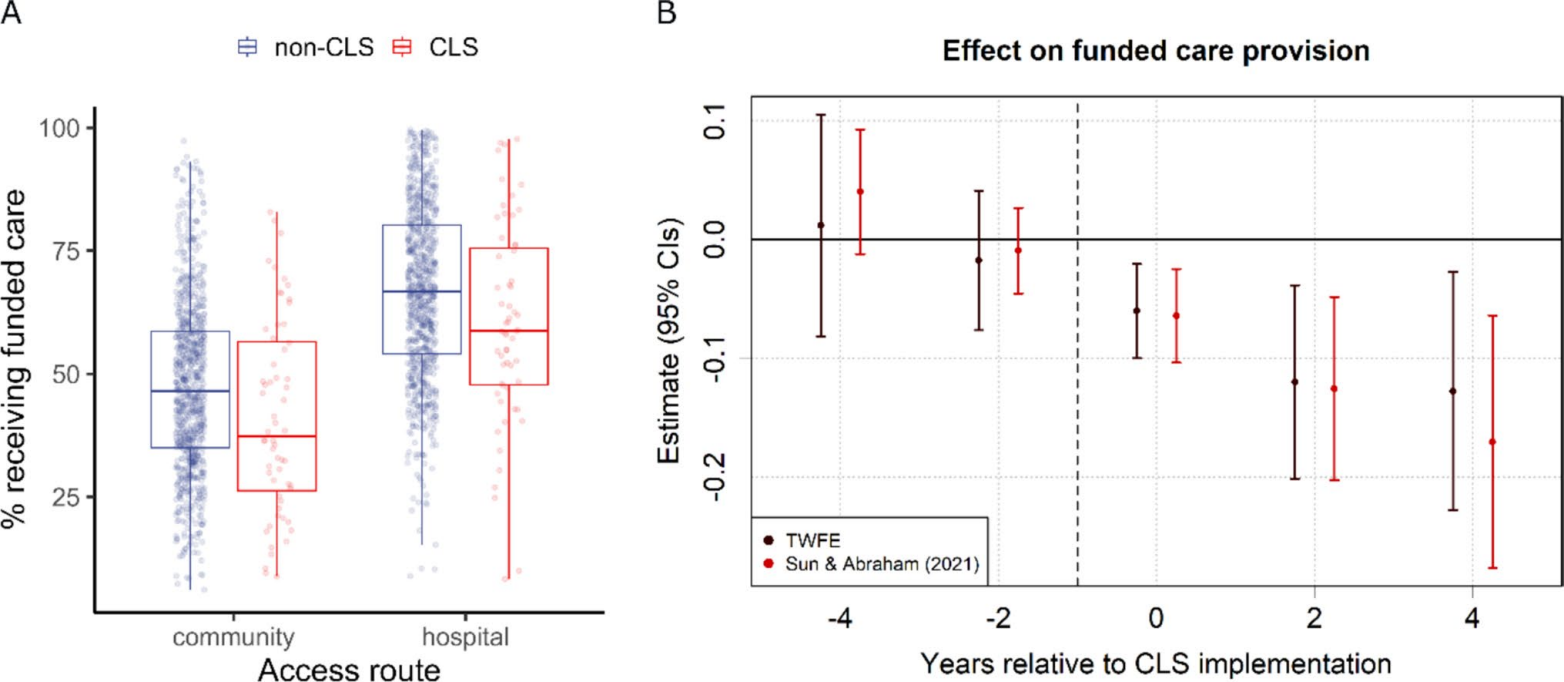
Community led support (CLS) and the percentage of requests receiving funded care. **A** Boxplots representing the unadjusted distribution of values for CLS (red) and Non-CLS (blue) years within local authorities in England from 2016 to 2021, divided by Access Route. **B** CLS coefficients and 95% confidence intervals (CIs) for time periods pre- and post-implementation (reference year = − 1), computed using two-way fixed effects (TWFE) and Sun and Abraham (2021) methods

**Fig. 2:**
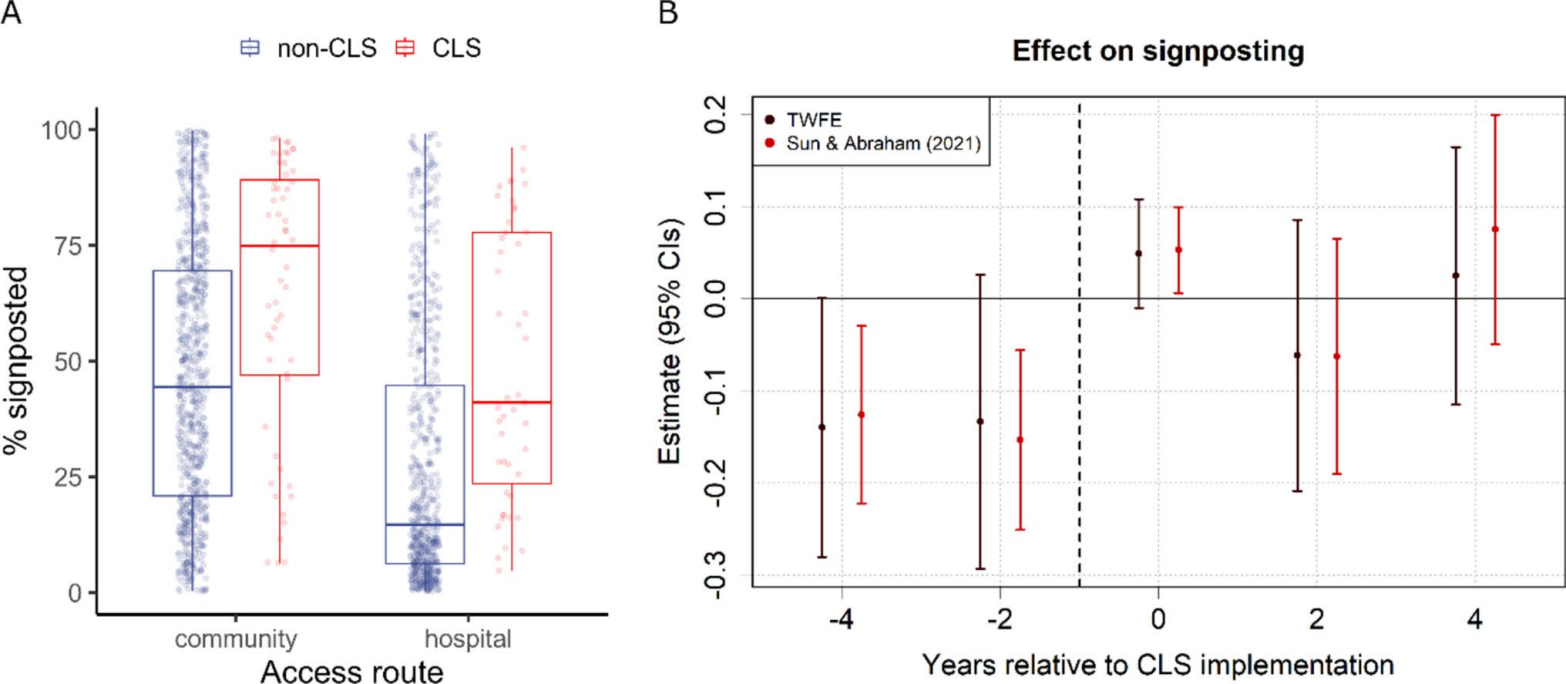
Community led support (CLS) and the percentage of non-funded requests that were signposted to other services. **A** Boxplots representing the unadjusted distribution of values for CLS (red) and non-CLS (blue) years within local authorities in England from 2016 to 2021, divided by Access Route. **B** CLS coefficients and 95% confidence intervals (CIs) for time periods pre- and post-implementation (reference year = -1), computed using two-way fixed effects (TWFE) and Sun and Abraham (2021) methods

Our analysis on care provision focused on how CLS implementation affected the percentage of care requests that resulted in funded care (Fig. 1) and the percentage of non-funded care requests that were signposted to other services (Fig. 2). Figure 1a plots the percentage of funded requests for CLS sites’ post-implementation years in red, with all other ‘non-CLS’ years plotted in blue. Boxplots overlay the individual values and illustrate the change in funded care following CLS implementation (*M*_*diff*_ = − 6.5%). The results of a DiD event study are summarised in Table 2 and displayed in Fig. 1b, estimating the treatment effect of CLS on funded care.

**Table 2:** Funded care—proportion of total requests

	TWFE			Sun and Abraham (2021)		
*Parameter*	$\widehat{\beta }$(*SE*_*CR*_)	*Conf. intervals*		$\widehat{\beta }$(*SE*_*CR*_)	*Conf. intervals*	
		*lower*	*upper*		*lower*	*upper*
CLS (ATT)	− 0.10 (0.03)***	− 0.15	− 0.04	− 0.10 (0.02)***	− 0.14	− 0.05
CLS (year -4)	0.01 (0.05)	− 0.08	0.11	0.04 (0.03)	− 0.01	0.09
CLS (year -2)	− 0.02 (0.03)	− 0.08	0.04	− 0.009 (0.02)	− 0.05	0.03
CLS (year 0)	− 0.06 (0.02)**	− 0.10	− 0.02	− 0.06 (0.02)**	− 0.10	− 0.02
CLS (year 2)	− 0.12 (0.04)**	− 0.20	− 0.04	− 0.13 (0.04)**	− 0.20	− 0.05
CLS (year 4)	− 0.13 (0.05)*	− 0.23	− 0.03	− 0.17 (0.05)**	− 0.28	− 0.06
	*N*_*obs*_ = 1769, *R*^*2*^ = 0.603			*N*_*obs*_ = 1768, *R*^*2*^ = 0.606		

+ *p* < .1, * *p* < .05, ** *p* < .01, *** *p* < .001. Estimates, cluster-robust standard errors and 95% confidence intervals for models predicting the effect of CLS implementation on the proportion of total requests receiving funded care. The first model uses a conventional two-way fixed effects (TWFE) difference-in-difference approach, while the second uses the Sun and Abraham (2021) method for dynamic treatment effects. CLS parameters are displayed for five time periods pre- and post-implementation, using the year prior to implementation (year -1) as the reference level. The average treatment effect on the treated (ATT) was calculated by averaging across the post-implementation coefficients. The model includes all variables in Table 1 as controls

The average treatment effect (ATT) of CLS on care provision (TWFE: $\widehat{\beta }$ = − 0.095, *t* = 3.53, *p* < 0.001; S&A: $\widehat{\beta }$ = − 0.096, *t* = 4.39, *p* < 0.001) indicated a reduction in funded care equivalent to 10 percentage points following CLS implementation. Consistent with a causal effect of CLS, pre-implementation time periods did not significantly differ from the reference year, while all three time periods post-implementation showed significant reductions in the proportion of funded care (see Fig. 1b). Moreover, CLS effects scaled with the number of years post-implementation, from 6 percentage points in the first time period increasing to 13 and 17 percentage points in the second (year 2) and third (year 4) time periods, respectively (S&A estimates).

To investigate the parallel trends assumption, we first conducted a TWFE event study to visualise year-by-year effects of CLS pre- and post-treatment (Appendix Fig. 5). We then conducted a placebo test, setting the CLS intervention time point four years earlier. Aside from a borderline effect in the second year post-sham intervention (*p* = 0.049), there were no effects of CLS pre-implementation (see Fig. 5). To confirm this, we conducted a pre-trend test, finding that the aggregated pre-treatment coefficients were not significantly different from zero (pre-trend ATT = 0.003, *t* = 0.10, *p* = 0.922). Further, we estimated the robustness of the CLS treatment effect by varying the sampling window (3, 4, and 5 years pre- and post-implementation). We also computed doubly robust DiD estimates that relax the assumption of homogenous treatment effect across covariates (Callaway & Sant’Anna 2021). These results are provided in Appendix Tables 10 and 11 and align with our main findings.

### Signposting to other services

Of those requests that did not receive funded care, we found that, in general, CLS did not increase the proportion signposted to other services (TWFE: $\widehat{\beta }$ = 0.004, *t* = 0.10, *p* = 0.923; S&A: $\widehat{\beta }$ = 0.013, *t* = 0.40, *p* = 0.689), relative to the year prior to its implementation (see Table 3 and Fig. 2), though we do note a five-percentage-point uptick in the first time period post-implementation.

**Table 3:** Signposted—proportion of non-funded requests

	TWFE			Sun and Abraham (2021)		
*Parameter*	$\widehat{\beta }$(*SE*_*CR*_)	*Conf. intervals*		$\widehat{\beta }$(*SE*_*CR*_)	*Conf. intervals*	
		*lower*	*upper*		*lower*	*upper*
CLS (ATT)	0.004 (0.05)	− 0.09	0.10	0.01 (0.03)	− 0.05	0.08
CLS (year -4)	− 0.14 (0.07) +	− 0.28	0.002	− 0.13 (0.05)*	− 0.22	− 0.03
CLS (year -2)	− 0.13 (0.08)	− 0.29	0.03	− 0.15 (0.05)**	− 0.25	− 0.06
CLS (year 0)	0.05 (0.03)	− 0.01	0.11	0.05 (0.02)*	0.006	0.10
CLS (year 2)	− 0.06 (0.07)	− 0.21	0.09	− 0.06 (0.06)	− 0.19	0.07
CLS (year 4)	0.02 (0.07)	− 0.12	0.17	0.07 (0.06)	− 0.05	0.20
	*N*_*obs*_ = 1563, *R*^*2*^ = 0.625, *AIC* = − 525			*N*_*obs*_ = 1562, *R*^*2*^ = 0.629, *AIC* = − 522		

+ *p* < .1, * *p* < .05, ** *p* < .01, *** *p* < .001. Estimates, cluster-robust standard errors and 95% confidence intervals for models predicting the effect of CLS implementation on the proportion of non-funded requests signposted to other services. The first model uses a conventional two-way fixed effects (TWFE) difference-in-difference approach, while the second uses the Sun and Abraham (2021) method for dynamic treatment effects. CLS parameters are displayed for five time periods pre- and post-implementation, using the year prior to implementation (year -1) as the reference level. The average treatment effect on the treated (ATT) was calculated by averaging across post-implementation coefficients. The model includes all variables in Table 1 as controls

The pre-intervention effect of CLS was non-significant (TWFE pre-trend ATT = -0.132, *t* = 1.83, *p* = 0.067). This indicates that the assumption of parallel trends was not violated and that overall levels of signposting did not differ between CLS and non-CLS sites pre-implementation. The placebo test findings (Appendix Fig. 6﻿) corroborate this result. Results from the variable sampling (Apendix Table 12) analysis also indicate no effect of CLS on signposting.

### Care reviews

**Fig. 3:**
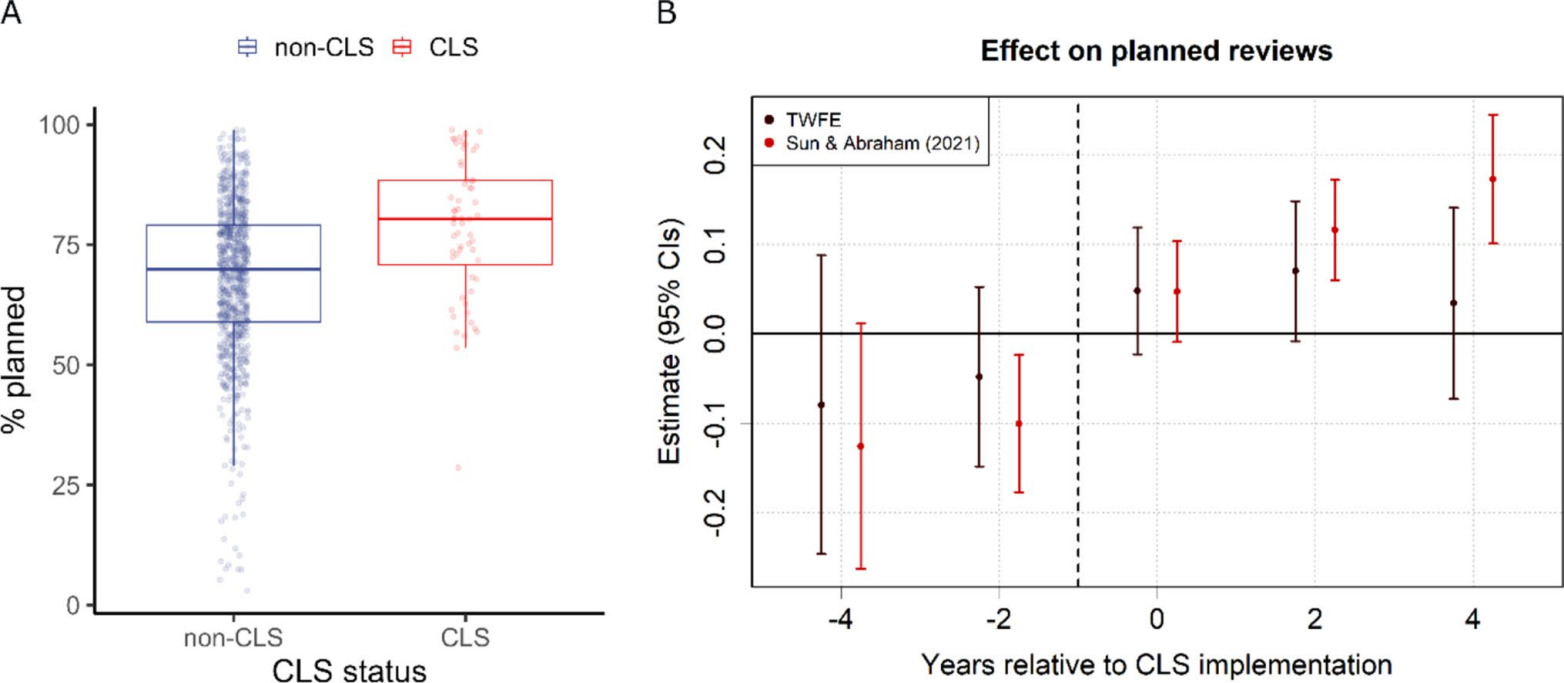
Community led support (CLS) and the percentage of planned reviews for existing clients. **A** Boxplots representing the unadjusted distribution of values for CLS (red) and non-CLS (blue) years within local authorities in England from 2016 to 2022. **B** CLS coefficients and 95% confidence intervals (CIs) for time periods pre- and post-implementation (reference year = − 1), computed using two-way fixed effects (TWFE) and Sun and Abraham (2021) methods

Next, we assessed the impact of CLS on existing clients. The percentage of reviews that were planned (as opposed to unplanned) are plotted for CLS and non-CLS years in Fig. 3a. Table 4 summarises the results of the DiD analysis, and the parameters for CLS are plotted in Fig. 3b.

**Table 4:** Planned reviews—proportion of total reviews

	TWFE			Sun and Abraham (2021)		
*Parameter*	$\widehat{\beta }$(*SE*_*CR*_)	*Conf. intervals*		$\widehat{\beta }$(*SE*_*CR*_)	*Conf. intervals*	
		*lower*	*upper*		*lower*	*upper*
CLS (ATT)	0.05 (0.04)	− 0.02	0.12	0.08 (0.03)**	0.03	0.13
CLS (year -4)	− 0.08 (0.08)	− 0.25	0.09	− 0.13 (0.08) +	− 0.26	0.01
CLS (year -2)	− 0.05 (0.05)	− 0.15	0.05	− 0.10 (0.05)*	− 0.18	− 0.02
CLS (year 0)	0.05 (0.04)	− 0.02	0.12	0.05 (0.04)	− 0.009	0.10
CLS (year 2)	0.07 (0.04) +	− 0.009	0.15	0.12 (0.04)***	0.06	0.17
CLS (year 4)	0.03 (0.05)	− 0.07	0.14	0.17 (0.04)***	0.10	0.25
	*N*_*obs*_ = 891, *R*^*2*^ = 0.698, *AIC* = − 1451			*N*_*obs*_ = 890, *R*^*2*^ = 0.704, *AIC* = − 1446		

+ *p* < .1, * *p* < .05, ** *p* < .01, *** *p* < .001. Estimates, cluster-robust standard errors and 95% confidence intervals for models predicting the effect of CLS implementation on the proportion of total reviews that were planned. The first model uses a conventional two-way fixed effects (TWFE) difference-in-difference approach, while the second uses the Sun and Abraham (2021) method for dynamic treatment effects. CLS parameters are displayed for five time periods pre- and post-implementation, using the year prior to implementation (year -1) as the reference level. The average treatment effect on the treated (ATT) was calculated by averaging across post-implementation coefficients. The model includes all variables in Table 1 as controls

The results from Sun and Abrahams (2021) model indicate that the CLS programme increased the proportion of planned care reviews by 8 percentage points overall, $\widehat{\beta }$ = 0.082, *t* = 3.08, *p* = 0.002, though the average treatment effect using two-way fixed effects estimation was non-significant, $\widehat{\beta }$ = 0.053, *t* = 1.49, *p* = 0.139. The S&A model also found increasing effects of CLS across time points, including increases in planned reviews of 12 and 17 percentage points for years 2 and 4 post-implementation, respectively.

A pre-trend test indicated that the overall level of planned reviews did not differ between CLS and non-CLS sites prior to implementation (TWFE pre-trend ATT = -0.06, *t* = 1.02, *p* = 0.306). Our placebo test did reveal a dip in the proportion of planned reviews in years -3 and -4 (see Appendix Fig. 7). However, this likely does not reflect a linear trend given the lack of differences in other years (particularly year -5) and overall (see Table 11). Our sample analysis (Appendix Table 13) and the doubly robust estimates (Table 11) suggest a positive effect of CLS on planned reviews, particularly in later time periods post-implementation.

### Care expenditure

**Fig. 4:**
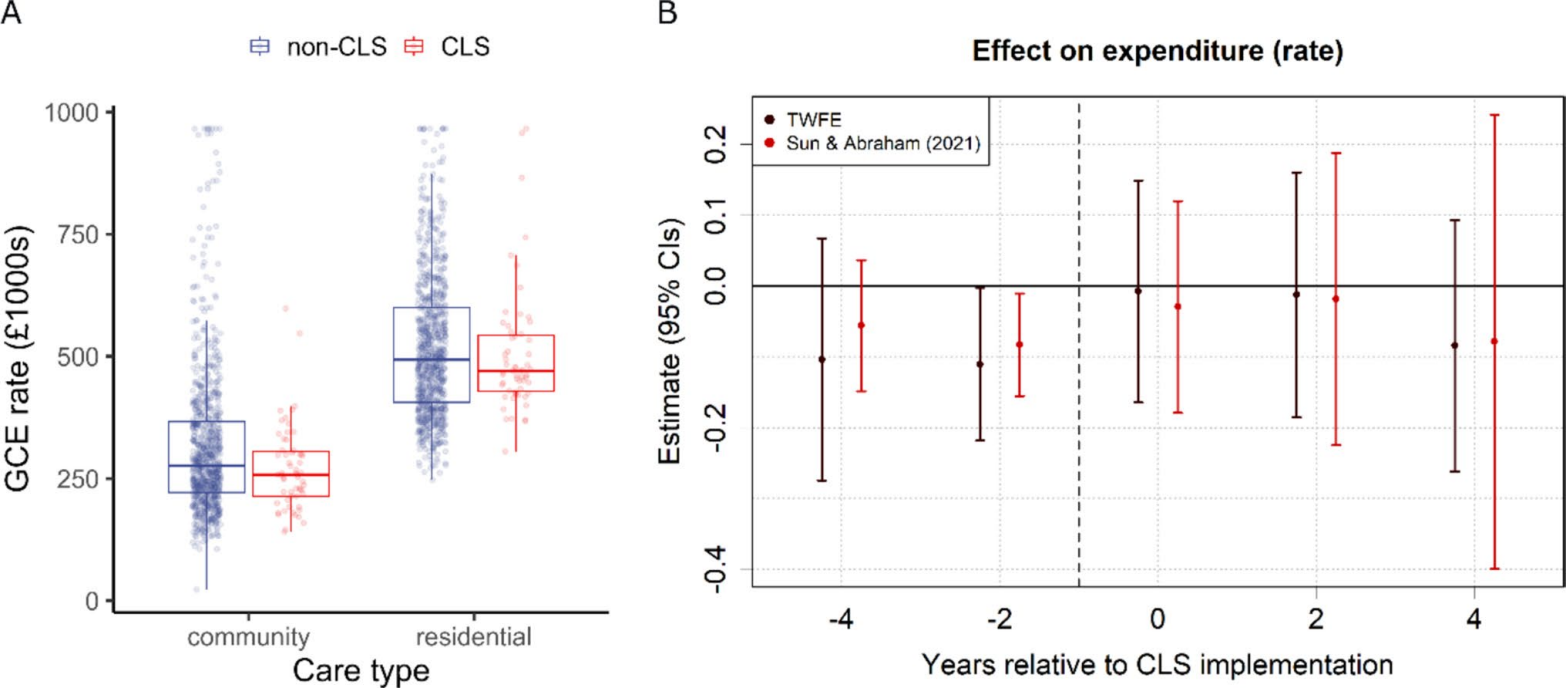
Community led support (CLS) and the expenditure rate per 1000 residents over 65 (GCE: gross current expenditure in £1000 s). **A** Boxplots representing the unadjusted distribution of values for CLS (red) and non-CLS (blue) years within local authorities in England from 2016 to 2022, divided by long-term care setting (community or residential care). **B** CLS coefficients and 95% confidence intervals (CIs) for time periods pre- and post-implementation (reference year = − 1), computed using two-way fixed effects (TWFE) and Sun and Abraham (2021) methods

**Fig. 5:**
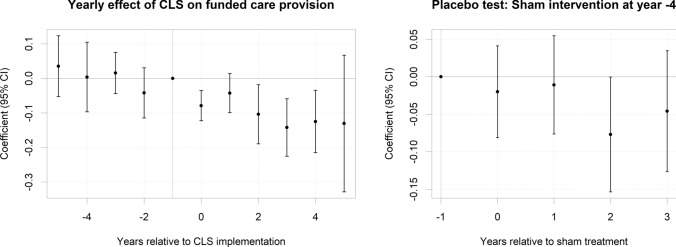
Yearly effect of CLS on funded care provision (left) across five years pre- and post-implementation (reference year = -1), using two-way fixed effects (TWFE). As well as the results of a placebo test with a sham CLS intervention at year − 4 to test the parallel trends assumption (right)

Finally, we investigated whether implementing CLS affected the rate of local authority spending on long-term care. Figure 4a displays gross current expenditure (GCE) per 1000 residents over 65 years of age (£1000 s), separated by long-term care setting (Care Type: community or residential) and CLS status. As in previous sections, we used two fixed effects modelling approaches (TWFE and S&A) to estimate the effect of CLS, this time on the log rate of GCE per 1000 residents over 65 (Table 5 and Fig. 4b).

**Table 5:** Expenditure—log-rate GCE per 1000 residents over 65

	TWFE			Sun and Abraham (2021)		
*Parameter*	$\widehat{\beta }$(*SE*_*CR*_)	*Conf. intervals*		$\widehat{\beta }$(*SE*_*CR*_)	*Conf. intervals*	
		*lower*	*upper*		*lower*	*upper*
CLS (ATT)	− 0.03 (0.08)	− 0.18	0.13	− 0.03 (0.09)	− 0.21	0.15
CLS (year -4)	− 0.10 (0.09)	− 0.28	0.07	− 0.06 (0.05)	− 0.15	0.04
CLS (year -2)	− 0.11 (0.06)*	− 0.22	− 0.002	− 0.08 (0.04)*	− 0.16	− 0.01
CLS (year 0)	− 0.007 (0.08)	− 0.17	0.15	− 0.03 (0.08)	− 0.18	0.12
CLS (year 2)	− 0.01 (0.09)	− 0.19	0.16	− 0.02 (0.11)	− 0.23	0.19
CLS (year 4)	− 0.08 (0.09)	− 0.26	0.10	− 0.08 (0.16)	− 0.40	0.24
	*N*_*obs*_ = 1780, *R*^*2*^ = 0.704, *AIC* = 328			*N*_*obs*_ = 1779, *R*^*2*^ = 0.705, *AIC* = 340		

+ *p* < .1, * *p* < .05, ** *p* < .01, *** *p* < .001. Estimates, cluster-robust standard errors and 95% confidence intervals for models predicting the effect of CLS implementation on the log rate of GCE (gross current expenditure) per 1000 residents over 65 years of age. The first model uses a conventional two-way fixed effects (TWFE) difference-in-difference approach, while the second uses the Sun and Abraham (2021) method for dynamic treatment effects. CLS parameters are displayed for five time periods pre- and post-implementation, using the year prior to implementation (year -1) as the reference level. The average treatment effect on the treated (ATT) was calculated by averaging across post-implementation coefficients. The model includes all variables in Table 1 as controls

Our analysis found no overall effect of CLS on care expenditure (GCE) rates (TWFE: $\widehat{\beta }$ = − 0.026, *t* = 0.33, *p* = 0.740; S&A: $\widehat{\beta }$ = − 0.031, *t* = 0.33, *p* = 0.741), and no effect for any of the post-implementation time-period coefficients. The pre-trend test (TWFE pre-trend ATT = -0.07, *t* = 1.15, *p* = 0.250), and placebo test (Appendix Fig. 8) show no evidence of a CLS effect prior to implementation (see also Appendix Tables 11 and 14).

## Discussion

Despite its popularity, evidence supporting the effectiveness of strengths-based approaches in adult social care is limited, partly due to a lack of consensus in how to measure and evaluate its impact (Caiels et al. 2021). In this paper, we estimate the effects of community led support (CLS), which is a strengths-based programme funded by NDTI and implemented in 16 local authorities across England (Brown et al. 2020; Richardson & Pitts 2023). Although flexible in its implementation, CLS expects to provide outcomes aligned with its core principles (Table 6) with commonality between the participating sites in their assumptions regarding the benefits of the programme and how it would change practitioner behaviour and organisational systems. In the current analysis, we used these expected outcomes to hypothesise differences that should be visible in SALT administrative data (2016–2021) after the programme has been implemented.

Firstly, given the emphasis in CLS on utilising local, community-based resources, we hypothesised that CLS implementation would lead to fewer people receiving funded care, and that a greater proportion of those not receiving care would be signposted to alternative community-based care options. This prediction was supported by the data, in part. CLS implementation led to a ten-percentage-point reduction in funded care provision overall, with later time periods post-implementation seeing the largest reductions. However, we found little evidence of increased signposting. Overall, our findings suggest that CLS has had a significant impact on care pathways within implementation sites, diverting more people away from ‘traditional’ local authority funded care, but it is unclear from our data whether those not receiving care were signposted to alternative sources of support. It could be the case that a significant proportion of CLS signposting occurred outside of the official requests for care (e.g., at community hubs), and are not captured in the SALT dataset, meaning they are missed in our analysis. Though we found no evidence that CLS sites differed from non-CLS sites on regional covariates, it is plausible that reductions in funded care might reflect general cost-cutting in the same time frame.

Next, we hypothesised that greater CLS-driven efficiencies in the care system would lead to improved care for existing clients, but without increases in spending. As a general indicator of quality of practice, we considered care reviews, reasoning that a higher proportion of planned to unplanned reviews would indicate a more preventative, rather than reactionary, style of care. Using the Sun and Abraham (2021) estimation approach, we found that the proportion of planned reviews was eight percentage points higher following CLS implementation, suggesting that long-term clients within those sites were receiving higher quality care, at least according to this metric. The effect was non-significant within the first period post-implementation, but was present and increasing after the programme had been established for two years. There was also no significant difference in the rate of local authority spending following CLS implementation. Taken together, these findings suggest that care improvements were realised without an increase in expenditure.

CLS has therefore had an impact on both new and existing clients, but the question of *how* CLS achieved these outcomes is more complex and beyond the scope of this current work. Supporters of CLS and strengths-based models more broadly accept that implementation strategies will likely differ from place to place (Brown et al. 2017, 2020; Miller & Whitehead 2015; Richardson & Pitts 2023). This is often seen as a strength, as a broad and flexible framework can be adapted to meet the specific needs of a local region, but it also leads to challenges in evaluating which aspects of the model are working and why (Caiels et al. 2021; Daly & Westwood 2018; Staudt et al. 2001). Future work should identify the mechanisms of change within strengths-based practice, to better guide its implementation, and to help deliver on desired outcomes. In our theory of change (Miller et al. 2024), participating sites saw key opportunities as being the introduction of more flexible processes, which provided practitioners greater opportunity to support people based around their informal supports and community resources, as well as the training, development, and professional supervision which gave them the skills and confidence to practice more creatively.

In the current analysis, we have taken steps to minimise bias in our estimates of the impact of CLS—for instance, using a dynamic difference-in-difference framework (Sun & Abraham 2021)—but we also note some limitations. Firstly, the nature of strengths-based approaches, CLS included, leads to differences in how the framework is implemented. Here, we have focused on the core principles of CLS and have tested hypotheses that are based on outcomes that are expected across all sites, regardless of specific implementation strategies (Table 6). However, such heterogeneity may limit the generalisation of our results to specific regional models of CLS. Secondly, we note that local authorities may implement alternative care initiatives alongside CLS, including other forms of strengths-based practice. Although the DiD approach was selected to isolate the causal impact of CLS within specific regions and time points, we also recognise the complexity inherent within the systems of care that we are studying. Finally, we note that these results reflect the implementation of CLS in England and may not generalise to other countries.

In this paper, we have taken a wide-lensed approach, investigating whether effects of strengths-based care initiatives—in this case, community led support—are visible at the national level. Although the current work was focused on CLS specifically, we believe these findings could contribute towards an evidence base for strengths-based practice more generally, and will have implications for policymakers and practitioners working in that field.

## Figures and Tables

**Fig. 6:**
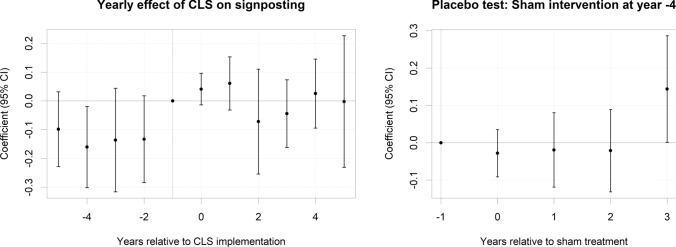
Yearly effect of CLS on signposting (left) across five years pre- and post-implementation (reference year = − 1), using two-way fixed effects (TWFE). As well as the results of a placebo test with a sham CLS intervention at year − 4 to test the parallel trends assumption (right)

**Fig. 7:**
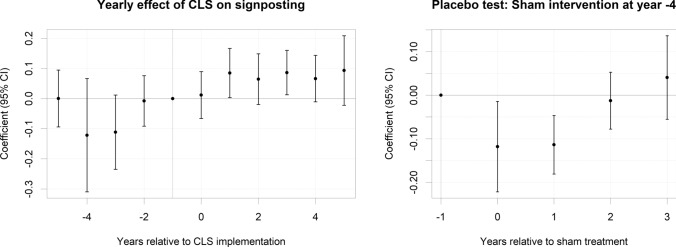
Yearly effect of CLS on planned reviews (left) across five years pre- and post-implementation (reference year = − 1), using two-way fixed effects (TWFE). As well as the results of a placebo test with a sham CLS intervention at year − 4 to test the parallel trends assumption (right)

**Fig. 8:**
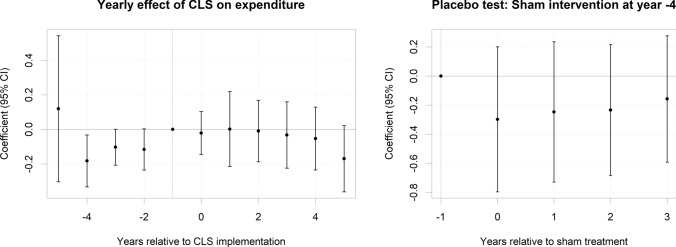
Yearly effect of CLS on rates of expenditure (left) across five years pre- and post-implementation (reference year = − 1), using two-way fixed effects (TWFE). As well as the results of a placebo test with a sham CLS intervention at year − 4 to test the parallel trends assumption (right)

## Data Availability

No datasets were generated or analysed during the current study.

## Appendix

See Figs. 5, 6, 7, 8.

See Tables

**Table 6:** Outcomes from community led support (CLS)

	Outcome
1	Easier, quicker access to the right support for each person
2	An increased presence and use of local solutions and options for support
3	Local services help people achieve their goals, and the feel happier and more resilient
4	Local services offer holistic, seamless support (as a result of changes introduced)
5	Staff delivering CLS are empowered and confident
6	Better use of local resources (value for money, efficiencies, effectiveness)

*Reproduced from* Brown et al., (2020)

6,

**Table 7:** Community led support (CLS) sites

*CLS Site*	*Year Established*
Barnsley	2021–22
Bradford	2017–18
Cheshire West and Chester	2021–22
Doncaster	2016–17
Hartlepool	2019–20
Leeds	2014–15
Salford	2019–20
Somerset	2015–16
Stoke-on-Trent	2020–21
Swindon	2020–21
Thurrock	2017–18
Torbay	2018–19
Warrington	2020–21
Warwickshire	2017–18
West Sussex	2018–19
York	2017–18

*Year Established’ refers to the financial year (March—April)*

7,

**Table 8:** Signposted—proportion of non-funded requests (log-odds)

	TWFE			Sun and Abraham (2021)		
*Parameter*	$\widehat{\beta }$(*SE*_*CR*_)	*Conf. intervals*		$\widehat{\beta }$(*SE*_*CR*_)	*Conf. intervals*	
		*lower*	*upper*		*lower*	*upper*
CLS (ATT)	− 0.01 (0.26)	− 0.52	0.50	0.05 (0.19)	− 0.32	0.41
CLS (year -4)	− 0.72 (0.34)*	− 1.39	− 0.05	− 0.60 (0.29)*	− 1.16	− 0.04
CLS (year -2)	− 0.71 (0.39) +	− 1.46	0.05	− 0.75 (0.24)**	− 1.23	− 0.28
CLS (year 0)	0.24 (0.16)	− 0.07	0.55	0.25 (0.12)*	0.008	0.49
CLS (year 2)	− 0.37 (0.42)	− 1.19	0.44	− 0.35 (0.36)	− 1.06	0.35
CLS (year 4)	0.09 (0.42)	− 0.73	0.91	0.40 (0.38)	− 0.34	1.13
	*N*_*obs*_ = 1563, *AIC* = 1648			*N*_*obs*_ = 1562, *AIC* = 1664		

+ *p* < .1, * *p* < .05, ** *p* < .01, *** *p* < .001. The models presented in Table 3 predicting the proportion of non-funded requests signposted to other services were re-estimated using general linear models with a logit-link. Estimates, cluster-robust standard errors and 95% confidence intervals for these models are presented here. The first model uses a conventional two-way fixed effects (TWFE) difference-in-difference approach, while the second uses the Sun and Abraham (2021) method for dynamic treatment effects. CLS parameters are displayed for five time periods pre- and post-implementation, using the year prior to implementation (year -1) as the reference level. The average treatment effect on the treated (ATT) was calculated by aggregating across post-implementation coefficients. The model includes all variables in Table 1 as controls

8,

**Table 9:** Comparison of CLS and non-CLS characteristics

	*CLS*	*Non-CLS*	*T-test P value*	*Logistic regression coefficient*
	*Mean (SD)*	*Mean (SD)*		
Carer's Allowance	3.47 (0.09)	3.40 (0.03)	0.46	-3.64 + (2.08)
Care bed availability	47.39 (2.56)	44.57 (0.92)	0.36	-0.03 (0.04)
Pension Credit	4.97 (0.08)	5.13 (0.03)	0.15	4.61 + (2.36)
Population size	3.90 (0.17)	3.93 (0.06)	0.91	0.60 (0.43)
Percentage over 80	26.73 (0.41)	26.94 (0.16)	0.68	-0.07 (0.18)
N	13	133		146

+ *p* < .1, * *p* < .05, ** *p* < .01, *** *p* < .001

^9^,

**Table 10:** Effect of CLS on funded care provision across different time samples

*Post-implementation year*	± *3 years*	± *4 years*	± *5 years*
CLS (year 0)	-0.076*** (0.022)	-0.077*** (0.022)	-0.079*** (0.022)
CLS (year 1)	-0.046 (0.028)	-0.044 (0.029)	-0.042 (0.029)
CLS (year 2)	-0.098* (0.042)	-0.104* (0.043)	-0.104* (0.043)
CLS (year 3)	-0.136** (0.042)	-0.142*** (0.042)	-0.142*** (0.042)
CLS (year 4)		-0.124** (0.046)	-0.125** (0.046)
	*N*_*obs*_ = 1729, *R*^*2*^ = 0.606	*N*_*obs*_ = 1753, *R*^*2*^ = 0.605	*N*_*obs*_ = 1763, *R*^*2*^ = 0.603

+ *p* < .1, * *p* < .05, ** *p* < .01, *** *p* < .001. Two-way fixed effect estimates for the treatment effect of CLS on the proportion of total requests receiving funded care for the first four years post-implementation. Estimates and cluster-robust standard errors are provided for different time samples, including 3, 4, and 5 years pre- and post-implementation. The model includes all variables in Table 1 as controls

10,

**Table 11:** Sensitivity analysis using doubly robust DiD

	Funded care	Signposted	Planned reviews	Care expenditure
Pre-intervention average effects (T < 0)	-0.02 (0.02)	0.03 (0.02)	0.01 (0.02)	-0.01 (0.08)
Pre-trend joint test (${\chi }^{2}\left(10\right)$)	253.87***	208.40***	34.76***	77.45***
T = -5	0.01 (0.03)	-0.07^+^ (0.04)	-0.11^+^ (0.06)	-0.20 (0.32)
T = -4	-0.02 (0.03)	-0.002 (0.06)	-0.01 (0.03)	0.08^+^ (0.05)
T = -3	-0.07** (0.03)	-0.02 (0.02)	0.07* (0.03)	-0.02 (0.03)
T = -2	0.02 (0.04)	0.21* (0.08)	0.06* (0.03)	0.08^+^ (0.04)
ATT (T > = 0)	-0.06 + (0.03)	0.02 (0.04)	0.05 (0.04)	-0.10 (0.09)
T = 0	-0.06** (0.02)	0.04 (0.04)	0.02 (0.05)	-0.04 (0.06)
T = 1	-0.04 (0.03)	-0.07 (0.10)	0.08^+^ (0.05)	-0.02 (0.11)
T = 2	-0.08^+^ (0.04)	-0.03 (0.05)	0.07 (0.05)	-0.07 (0.10)
T = 3	-0.11* (0.05)	-0.03 (0.05)	0.08^+^ (0.05)	-0.09 (0.12)
T = 4	-0.08 (0.06)	0.02 (0.07)	0.09* (0.04)	-0.17 (0.12)
T = 5	0.01 (0.14)	0.10 (0.11)	0.04 (0.06)	-0.31** (0.12)
T = 6	-0.04 (0.12)	0.19 (0.13)	-0.23 (0.30)	-0.39* (0.17)
T = 7	0.21*** (0.02)	0.05 (0.03)	0.08*** (0.02)	-0.20*** (0.03)
N	1769	1549	891	1780

+ *p* < .1, * *p* < .05, ** *p* < .01, *** *p* < .001. While pre-trend joint test rejects the null hypothesis that all pre-treatment coefficients are 0, the average deviation across all pre-treatment periods was not statistically significant (pre-intervention average effects). Estimated ATT from the doubly robust DiD aligns with our main results. However, this method is not preferred owing to our limited sample size in each cohort, while Sun and Abaham’s (2021) approach is more flexible to combine cohorts with small sample sizes

11,

**Table 12:** Effect of CLS on signposting across different time samples

*Post-implementation year*	± *3 years*	± *4 years*	± *5 years*
CLS (year 0)	0.039 (0.028)	0.038 (0.028)	0.041 (0.028)
CLS (year 1)	0.058 (0.045)	0.061 (0.046)	0.061 (0.047)
CLS (year 2)	-0.073 (0.094)	-0.071 (0.092)	-0.072 (0.092)
CLS (year 3)	0.045 (0.061)	-0.042 (0.060)	-0.044 (0.059)
CLS (year 4)		0.027 (0.061)	0.026 (0.061)
	*N*_*obs*_ = 1526, *R*^*2*^ = 0.621	*N*_*obs*_ = 1547, *R*^*2*^ = 0.622	*N*_*obs*_ = 1557, *R*^*2*^ = 0.624

+ *p* < .1, * *p* < .05, ** *p* < .01, *** *p* < .001. Two-way fixed effect estimates for the treatment effect of CLS on the proportion of non-funded requests signposted to other services for the first four years post-implementation. Estimates and cluster-robust standard errors are provided for different time samples, including 3, 4, and 5 years pre- and post-implementation. The model includes all variables in Table 1 as controls

12,

**Table 13:** Effect of CLS on care reviews across different time samples

*Post-implementation year*	± *3 years*	± *4 years*	± *5 years*
CLS (year 0)	0.010 (0.040)	0.011 (0.039)	0.012 (0.039)
CLS (year 1)	0.081 + (0.042)	0.084* (0.041)	0.085* (0.041)
CLS (year 2)	0.066 (0.043)	0.066 (0.043)	0.065 (0.043)
CLS (year 3)	0.088* (0.038)	0.088* (0.037)	0.086* (0.037)
CLS (year 4)		0.068 + (0.039)	0.067 + (0.039)
	*N*_*obs*_ = 871, *R*^*2*^ = 0.713	*N*_*obs*_ = 883, *R*^*2*^ = 0.712	*N*_*obs*_ = 888, *R*^*2*^ = 0.712

+ *p* < .1, * *p* < .05, ** *p* < .01, *** *p* < .001. Two-way fixed effect estimates for the treatment effect of CLS on the proportion of total reviews that were planned for the first four years post-implementation. Estimates and cluster-robust standard errors are provided for different time samples, including 3, 4, and 5 years pre- and post-implementation. The model includes all variables in Table 1 as controls

13,

**Table 14:** Effect of CLS on care expenditure across different time samples

*Post-implementation year*	± *3 years*	± *4 years*	± *5 years*
CLS (year 0)	0.023 (0.063)	-0.023 (0.063)	-0.022 (0.063)
CLS (year 1)	-0.008 (0.109)	0.001 (0.110)	0.002 (0.110)
CLS (year 2)	-0.016 (0.092)	-0.007 (0.090)	-0.009 (0.090)
CLS (year 3)	0.040 (0.100)	-0.030 (0.097)	-0.032 (0.097)
CLS (year 4)		-0.051 (0.092)	-0.053 (0.092)
	*N*_*obs*_ = 1740, *R*^*2*^ = 0.704	*N*_*obs*_ = 1764, *R*^*2*^ = 0.705	*N*_*obs*_ = 1774, *R*^*2*^ = 0.705

+ *p* < .1, * *p* < .05, ** *p* < .01, *** *p* < .001. Two-way fixed effect estimates for the treatment effect of CLS on the log rate of GCE (gross current expenditure) per 1000 residents over 65 years of age for the first four years post-implementation. Estimates and cluster-robust standard errors are provided for different time samples, including 3, 4, and 5 years pre- and post-implementation. The model includes all variables in Table 1 as controls

14.
